# Hyperlactatemia in a group of HIV patients living in Yaounde-Cameroon

**DOI:** 10.1186/1742-6405-11-2

**Published:** 2014-01-15

**Authors:** Corinne Tchoula Mamiafo, Vicky Jocelyne Ama Moor, Jobert Richie N Nansseu, Constant Anatole Pieme, Claude Tayou, Jeanne Ngogang Yonkeu

**Affiliations:** 1 Laboratory of Biochemistry, Yaounde University Teaching Hospital, Yaounde, Cameroon; 2 Department of Biochemistry and Physiological Sciences, University of Yaounde I, Yaounde, Cameroon; 3 Department of Biochemistry, Faculty of Sciences, University of Yaounde I, Yaounde, Cameroon; 4 Intensive Care Unit, Mother and Child Centre, Chantal Biya Foundation, Yaounde, Cameroon; 5 Laboratory of Hematology, Yaounde University Teaching Hospital, Yaounde, Cameroon

**Keywords:** Hyperlactatemia, Antiretroviral therapy, HIV, First line regimen

## Abstract

**Background and aim:**

Prolonged exposure to highly active antiretroviral therapy (HAART) is associated with adverse effects such as hyperlactatemia. We determined the prevalence and risk factors for developing hyperlactatemia among human immunodeficiency virus (HIV)-infected cameroonians on antiretroviral therapy (ART).

**Methods:**

We conducted a cross-sectional study from January to April 2012 involving 91 HIV-infected patients receiving ART for at least 12 months and 30 HIV-infected patients who have never received ART (ART-naïve patients). Plasma lactate levels were determined after at least 12 hours of overnight fasting and hyperlactatemia defined as lactate concentrations ≥ 3 mmol/L. The prevalence of hyperlactatemia was determined and the risk factors were analyzed by a multivariate logistic regression model.

**Results:**

The mean lactataemia was significantly higher in the group of HIV patients currently taking ART than in the ART-naïve one (2.3 ± 1.3 and 1.7 ± 0.7 mmol/L respectively, p = 0.002). Patients on first line ART regimens had significantly higher lactatemia than those on second line regimens (2.5 ± 1.5 and 1.9 ± 0.7 mmol/L respectively, p = 0.014). The prevalence of hyperlactatemia in HIV patients receiving ART and in ART-naïve HIV patients was respectively 18.7 and 6.7% (p = 0.095). ART-exposure (adjusted odds ratio (aOR) 5.44, 95% confidence interval (CI) 1.06 – 27.84; p = 0.042) and being on a first line regimen (aOR 16.22, 95% CI 1.57 – 167.91; p = 0.019) were independent strong predictors of hyperlactatemia.

**Conclusion:**

Hyperlactatemia was not rare in our study population. Being on a first line regimen constitutes an important risk factor for developing hyperlactatemia. Measurement of plasma lactate may be useful in optimizing the management of HIV-positive persons on ART.

## Introduction

AIDS remains one of the major public health hazards in Africa. The advent and widespread use of HAART in clinical practice has profoundly modified the natural history of HIV infection by significantly improving the prognosis and quality of life of people living with the infection, with a drastic reduction in mortality and morbidity related to HIV and its complications [1]. Unfortunately, the need for permanent medication has led to increasingly more numerous descriptions of new adverse metabolic effects, this being explained by mitochondrial toxicity [2]. It has been demonstrated that this mitochondrial toxicity is associated with hyperlactatemia, lactic acidosis, hepatic steatosis, pancreatitis, lipodystrophy, and peripheral neuropathy [3].

Hyperlactatemia characterized by mild to moderate increases in blood lactate levels in the absence of acidosis has been reported in 8–36% of patients receiving HAART in developed countries, although the majority of cases are asymptomatic or have very mild symptoms [4-8]. Only a small number of patients develop the most severe form of hyperlactatemia, which presents as metabolic acidosis and is associated with a high mortality rate.

A variety of NRTIs, including stavudine, zidovudine and didanosine, have been associated with hyperlactatemia because of their potential for mitochondrial toxic effects [2,6,9-14]. While stavudine is rarely used in resource-rich settings and is no longer recommended by WHO for initial treatment of HIV-1 infection [14], it remains an important component of standard ART regimens in many resource-limited countries, mainly due to its lower cost [15,16]. Some observational studies have suggested that specific risk factors associated with the development of hyperlactatemia include female sex [17-23], elevated weight or BMI [17,20-23], older age (> 40 years) [17,20], and lower CD4 cell counts [17].

There are limited data on hyperlactatemia caused by prolonged exposure to ART in resource-limited settings like Cameroon. The present study sought at determining the prevalence and determinants related to the development of hyperlactatemia in HIV-infected Cameroonians currently on ART.

## Patients and methods

### Study participants

This cross-sectional survey was carried out at the HIV out-patient clinic of the Yaounde University Teaching Hospital and the Yaounde Central Hospital. These centers are located in the political capital of Cameroon, and receive a large number of HIV-infected patients living in Yaounde and surrounding town. A convenient sample of 91 HIV-infected patients aged 18 years and above , currently on ART for at least 12 months and regularly followed-up were consecutively enrolled in the study during January – April 2012. These patients were receiving either first line regimen consisting of 2 NRTIs + 1 NNRTI, or second line regimen comprising 2 NRTIs + 1 PI. The choice of a particular regimen was irrespective of potential factors that could induce hyperlactatemia but depended mainly on the availability of ARTs. For quality control purpose, a subset of 30 HIV-infected and ART-naïve patients was also recruited. All the participants were confirmed to be positive for HIV antibody through laboratory detection, and the diagnosis was in line with national HIV/AIDS diagnosis criteria. They were free of alcoholism, chronic hepatitis and were also required not to be on plasma lactate modifying therapies at their enrolment. All the procedures used in this study were in accordance with the current revision of the Helsinki Declaration. A Written and signed informed consent was provided by all the subjects. Consent forms and procedures, as well as survey protocol, were approved by the Cameroon National Ethics Committee (**Reference number: 234/CNE/SE/2011**).

### Interview data

At enrollment standardized data collection forms were completed, including sociodemographic characteristics, medical history (AIDS events, CDC/WHO clinical classification, signs and symptoms of symptomatic hyperlactatemia such as nonspecific gastrointestinal symptoms nausea, vomiting, abdominal pain, discomfort or distension, anorexia, peripheral neuropathy, tiredness, muscle weakness, and dyspnea [24]), laboratory markers (CD4 cell counts, FBS (Fasting Blood Sugar), AST (Aspartate Amino Transferase) and ALT (Alanine Amino Transferase) levels), and antiretroviral used.

### Sample collection and biochemical assays

After at least 12 hours of overnight fasting and a 30 minutes rest, blood was aseptically collected from each participant by venipuncture in a 5 ml EDTA tube, without a tourniquet or fist clenching. Samples were put on ice and immediately transported to the biochemistry laboratory where plasma specimens were separated by centrifugation at 3000 rpm within 5 min for analyses without delay. Plasma lactate concentrations were determined on an automated clinical chemistry analyzer (BA-88A, MINDRAY laboratory, China) using kits (BIOREX diagnostics, UK) based on the enzymatic conversion of L-lactate to pyruvate by L-lactate oxidase [25].

### End point definitions

Concentrations of plasma lactate less than 3 mmol/L were defined as normolactatemic, while those equal or above 3 mmol/L, were consider as hyperlactatemic.

### Statistical analysis

Data were coded, entered, and analyzed using SPSS version 20.0 (SPSS Inc., Chicago, Ilinois, USA). Qualitative and quantitative variables were analysed and compared with **χ**^**2**^ test and Student’s **t**-test respectively. Results are expressed as proportion or mean ± SD. The Pearson correlation was used to established the correlation between the variables. ORs with 95% CIs were used to appreciate the impact of different variables on the occurrence of hyperlactatemia, and were calculated by both univariate and multivariate logistic regression analyses while adjusting for confounders. A *p* value < 0.05 was used to characterize significant results.

## Results

**Figure 1:**
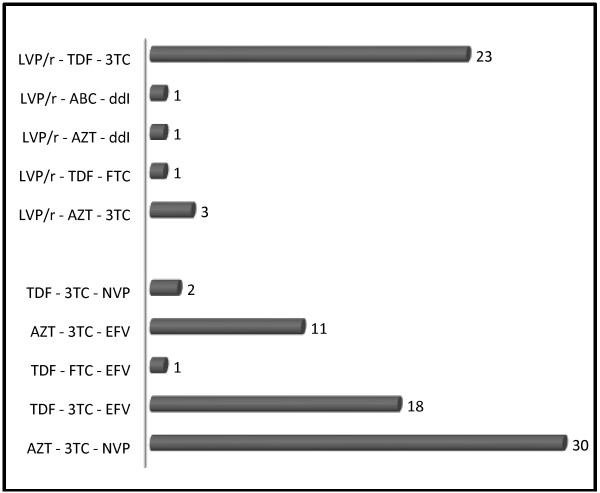
Different therapeutic regimens the 91 ART-receiving subjects were placed on: up are represented second line regimens and down the first line ones and the numbers refer to the number of subjects on each regimen.

A total of 121 patients were included in this study including 91 HIV patients currently taking ART, and 30 ART-naïve HIV patients. Table 1 presents the whole patient clinical characteristics and biochemical determinations. The age ranged from 25 to 67 years old (Mean = 40.3) for patients under ART and between 24 to 64 years old (Mean = 38) for naive ART patients. All the parameters recorded were higher in the group of patients on ART therapy compare to ART naïve except the level of LDH. There was no statistically significant difference between the two groups regarding all these parameters except lactatemia which was significantly (p < 0.005) higher in the group of patients on ART (Table 1). Only classes A and C of the CDC/WHO clinical classification of the HIV infection were noticed. Figure 1 depicts the different therapeutic regimens with the combination AZT – 3TC - NVP being the most recorded (32.97%). The first line ART regimens was the most encountered (66%). However 25.27% of the patients were on LVP/r- TDF-3TC (second line regimen). The mean lactataemia was significantly higher in the group of HIV patients currently taking ART than in the ART-naïve one (2.3 ± 1.3 and 1.7 ± 0.7 mmol/L respectively) (Table 1). Nineteen over the one hundred and twenty one participants (15.70%) presented with hyperlactatemia; among them 17 were HIV patients currently taking ART (18.7%) and the 2 others (6.7%) being ART-naïve patients (Table 1).

**Table 1:** Clinical and biochemical characteristics of the study participants

	**Mean ± SD**			**p**
	**Patients on ART (n = 91)**	**ART-naïve patients (n = 30)**	**Overall (n = 121)**	
**Age (years)**	40.3 ± 9.9	38.5 ± 11.8	39.8 ± 10.4	0.327
**Sex (Male/Female)**	22/69	6/24	28/93	0.638
**CDC Clinical Classification (A/C)**	90/1	30/0	120/1	0.752
**CD4 count (cells/mm**^**3**^**)**	410.4 ± 231.9	261.8 ± 173.0	373.2 ± 226.9	0.192
**AST (IU/L)**	27.8 ± 15.9	25.6 ± 10.1	27.3 ± 14.6	0.171
**ALT (IU/L)**	25.6 ± 20.8	23.1 ± 8.3	24.9 ± 18.4	0.104
**FPG (g/L)**	0.9 ± 0.2	0.9 ± 0.2	0.9 ± 0.2	0.564
**LDH (IU/L)**	441.1 ± 138.1	485.1 ± 168.6	452.0 ± 146.8	0.253
**Lactataemia (mmol/L)**	2.3 ± 1.3	1.7 ± 0.7	2.2 ± 1.2	0.002*
**Hyperlactatemic patients (%)**	18.7	6.7	15.7	0.095

FBS: Fasting Blood Sugar; ALT: Alanine Amino Transférase, AST: Aspartate Amino Transférase, LDH: Lactate Dehydrogenase; * p<0.05.

Table 2 compares clinical and biochemical parameters between normolactatemic and hyperlactatemic patients taking ART. We did not observe any statistically significant difference between the two groups according to age, sex, CD4+ T-cells count at time of hyperlactatemia, duration of cumulative ART exposure prior to hyperlactatemia, FBS, AST and ALT. On contrary, the mean serum LDH (Lactate Dehydrogenase), as well as lactatemia, were significantly higher in the hyperlactatemic group (p = 0.040 and p < 0.001 respectively). Sixteen patients (25.8%) on first line regimens significantly develop hyperlactatemia (p = 0.007). Only one patient on second line regimen (1.1%) presented with severe hyperlactatemia.

**Table 2:** Characteristics of patients on ART with regard to the occurrence of hyperlactatemia

	**Normolactatemic n = 74 (Mean ± SD)**	**Hyperlactatemic n = 17 (Mean ± SD)**	**p**
**Age (years)**	40.3 ± 9.4	39.9 ± 12.2	0.865
**Sex (Male/Female)**	16/58	6/11	0.189
**CD4 count at time of hyperlactatemia (cells/mm**^**3**^**)**	414.5 ± 230.8	392.8 ± 239.6	0.730
**Duration of cumulative ART exposure prior to hyperlactatemia (years)**	5.2 ± 2.9	4.5 ± 3.2	0.382
**ART regimens**			
**First line**	44	16	0.007*
**Second line**	30	1	
**CDC/WHO Class**			
**A**	73	17	0.813
**C**	1	0	
**FPG (g/L)**	0.9 ± 0.1	0.9 ± 0.2	0.220
**AST (IU/L)**	26.7 ± 12.0	32.8 ± 27.4	0.396
**ALT (IU/L)**	23.1 ± 11.4	36.6 ± 41.8	0.221
**LDH (IU/L)**	419.7 ± 107.9	534.3 ± 207.0	0.040*
**Lactataemia (mmol/L)**	1.8 ± 0.6	4.2 ± 1.8	< 0.001*

* p<0.05.

**Figure 2:**
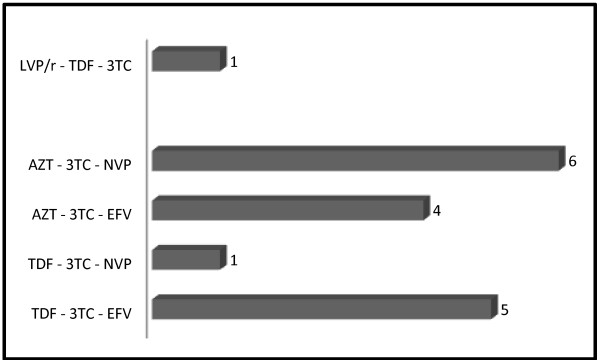
Regimens that have given rise to hyperlactatemia.

Figure 2 is representative of the regimens that have led to hyperlactatemia. There was no statistically significant difference when comparing the mean plasma lactate levels between these regimens. There was no correlation between lactatemia and age (r = 0.023, p = 0.825), CD4+ T-cells count (r = 0.071, p = 0.506), FBS (r = 0.151, p = 0.126), and between lactatemia and serum LDH (r = 0.181, p = 0.085). On the contrary, there was a positive and significant correlation between lactatemia and AST (r = 0.439, p < 0.001), and ALT (r = 0.544, p <0.001) (Table 3).

**Table 3:** Correlation between lactatemia and others parameters

**Variable**	**P**	**Pearson’s correlation coefficient (r)**
**Age**	0,825	0,023
**CD4 count**	0,506	0,071
**Fasting Plasma Glucose**	0,126	0,151
**LDH**	0,085	0,181
**AST**	<0,001*	**0,439**
**ALT**	<0,001*	**0,544**

* p<0.05.

While undertaking univariate logistic regression analyses, we did not find any statistically significant association between the occurrence of hyperlactatemia and the sex (OR 2.25, 95% CI 0.789 – 6.42; p = 0.123), the duration of cumulative ART exposure (OR 2.59, 95% CI 0.87 – 7.72; p = 0.089), and between the occurrence of hyperlactatemia and ART exposure (OR 3.22, 95% CI 0.70 – 14.93; p = 0.134) (Table 4). Nevertheless, this latter association became significant after adjusting for age, sex and duration of ART exposure with a coefficient of determination of 0.2 (aOR 5.44, 95% CI 1.06 – 27.84; p = 0.042), but after removing the duration of ART exposure from the model, the last-mentioned association was no more significant (aOR 4.09, 95% CI 0.82 – 20.33; p = 0.085). Being on a first line ART regimen increased more than 10 times the risk of developing hyperlactatemia (OR 10.91, 95% CI 1.37 – 86.70; p = 0.024), this risk becoming more important after adjusting for age, sex, and duration of ART exposure (aOR 16.22, 95% CI 1.57 – 167.91; p = 0.019) with a coefficient of determination of 0.32. We finally observe no significant association between the occurrence of hyperlactatemia and zidovudine-containing regimens, lamivudine-containing regimens, and with tenofovir-containing regimens (Table 4).

**Table 4:** Factors likely to influence the occurrence of hyperlactatemia

	**Odds ratio**	**95% CI**	**p**
**Sex**			
**Male**	1.00		
**Female**	2.25	0.79 – 6.42	0.130
**Age groups (years)**			
**20 – 29**	1.00		
**30 – 39**	0.42	0.07 – 2.57	0.347
**40 – 49**	0.67	0.11 – 4.15	0.664
**≥ 50**	0.21	0.02 – 2.84	0.243
**Duration of cumulative ART exposure**			
**< 5 years**	1.00		
**≥ 5 years**	2.59	0.87 – 7.72	0.089
**ART exposure**			
**No**	1.00		
**Yes**	3.22	0.70 – 14.93	0.134
**CDC clinical stage**			
**A**	1.19	1.1 – 1.28	0.843
**C**	1.00		
**ART regimen**			
**First line**	**10.91**	**1.37 – 86.70**	0.024*
**Second line**	1.00		
**First line AZT-regimens**			
**No**	1.00		
**Yes**	1.02	0.32 – 3.32	0.969
**First line TDF-regimens**			
**No**	1.00		
**Yes**	1.17	0.35 – 3.97	0.797
**First line 3TC-regimens**			
**No**	1.00		
**Yes**	0.98	0.94 – 1.02	0.742

* p<0.05.

## Discussion

Lactate is the final product of anaerobic glycolysis, which in a normal state does not accumulate in the body. However, under circumstances such as oxygen deprivation of tissue or impaired oxidative phosphorylation, hyperlactatemia occurs. Lactic acidosis in the absence of tissue ischemia is the hallmark of type B lactic acidosis [26]. Among all anti-HIV medications, it is the NRTIs that have been implicated in type B lactic acidosis [26]. In fact, the pharmacologically active triphosphate moieties of NRTIs act as substrates for human mitochondrial DNA-polymerase gamma, thereby having the potential for incorporation into mitochondrial DNA (mtDNA). As well, mitochondrial exonuclease is inefficient at removing the nucleoside triphosphates [27,28]. The combination of nucleoside triphosphate incorporation and inadequate removal disrupts mtDNA synthesis and results in the arresting of mtDNA-encoded protein synthesis. Eventually, a disruption of oxidative phosphorylation ensues, and lactate accumulates [2].

Stavudine, zidovudine and didanosine are the most frequent NRTIs incriminated in the occurrence of hyperlactatemia [2,6,9-14,29]. The combination of stavudine and didanosine is associated with the greatest relative risk [24]. In this study we had no patient placed on a stavudine-containing regimens because stavudine has been withdrawn from ART regimens recommended for anti-HIV medications in Cameroon since 2011, mainly due to its dyslipidemic effects proven in this milieu [30] and in accordance with the latest WHO guidelines [14]. We observed in our study that zidovudine, lamivudine and tenofovir were not implicated in the occurrence of hyperlactatemia, probably because of the small sample size and probably, the combination of all these nucleoside analogues would increase risk of adverse event development. These findings concur with those from Marceau et al. [29] where increased lactate was not associated with treatment containing lamivudine, zidovudine, abacavir, zalcitabine, efavirenz, nevirapine, and interferon and/or ribavirin. After regrouping regimens in first and second line regimen, we found lactatemia of patients on first line regimens being significantly higher than that of the ones on second line regimen. Furthermore, first line regimen use may confer a sixteen-fold increased risk of developing hyperlactatemia when controlling for other risk factors. These findings can be explained by the fact that PIs, which are part of second line regimen, have not been reported to cause any mitochondrial toxicity [7] given their lack of selectivity for HIV reverse transcriptase [14]. It has been also shown that the incidence and prevalence of hyperlactatemia may vary depending on the number and choice of NRTIs present in an antiretroviral regimen. In fact, in a longitudinal cohort study involving 2144 patients receiving NRTI therapy, the risk of symptomatic hyperlactatemia increased more than 2-fold for each additional NRTI used in a given regimen [31]. Different combinations of NRTIs were also associated with different rates of symptomatic hyperlactatemia [31].

We found significantly elevated plasma lactate levels in patients currently taking ART compared to ART-naïve ones, with an independent five-fold increased risk for the subjects on ART for developing hyperlactatemia when controlling for other covariates. But contrary to John et al. [7] we did not observe any relationship between the occurrence of hyperlactatemia and duration of cumulative ART exposure (especially stavudine in their case). The Lactic Acidosis International Study group [17], as well as Marceau et al. [32], showed an association of older age (age > 40 years) with the development of hyperlactatemia. This was not seen in our study nor in some other ones [7,29,33] probably because our study had a little bit younger age distribution than theirs (mean of 40 years vs. 42 and 44 years respectively). Moreover we found no relationship between the female sex and the occurrence of hyperlactatemia. The same results were reported in others studies [7,29,33]. By contrast, some relevant studies have clearly demonstrated that the female sex constitutes a strong independent risk factor for developing hyperlactatemia [17-20,22,23,34,35], even though the reasons for the observed sex differences in rates of toxicity remain uncertain. As shown in other studies, we observed no association between CD4+ T-cells count, CDC/WHO clinical classification and the occurrence of hyperlactatemia [7,29,33].

There is no consensus on the different thresholds for defining normolactatemia, mild to moderate hyperlactatemia (either symptomatic or not), and severe hyperlactatemia. It has been proposed, from a clinical point of view, that clinically relevant hyperlactatemia may be defined as a lactate concentration of 2.25 – 5 mmol/L, and severe lactic acidosis, as a lactate concentration > 5 mmol/L with a pH < 7.3 or bicarbonate < 20 mmol/L and clinical signs of multiorgan failure [36]. In their study, Marceau et al. [32], based on a previous determination of the reference interval for plasma lactate obtained from 200 healthy venous blood samples (range: 1.5 – 2.25 mmol/L; mean ± SD: 1.4 ± 0.3 mmol/L) [32], defined concentrations of plasma lactate < 2.25 mmol/L as normolactatemic, those between 2.25 and 5 mmol/L as moderately increased, and plasma lactate > 5 mmol/L was considered highly increased. With these definitions, 75.2% of their patients were normolactatemic, 23% had moderately increased serum lactate, and 1.8% had high serum lactate giving thus an overall prevalence of hyperlactatemia (plasma lactate ≥ 2.25 mmol/L) of 24.8% [32]. We had a little bit more normolactatemic subjects than them (81.3% vs. 75.2%) and less hyperlactatemic patients (18.7% vs. 24.8%) assumedly because our definition of normolactatemia was less restrictive than theirs (i.e. < 3 mmol/L vs. < 2.25 mmol/L). As we, they found that the mean value of plasma lactate was significantly higher in the hyperlactatemic group than in the normolactatemic one [32].

The prevalence of asymptomatic hyperlactatemia reported in resource-rich settings varies from one area to another 8.3% [33], 8.7% [37], 18.3% [7] and 21% [4]. As a matter of fact, the lack of a standard biochemical definition for lactic acidosis and symptomatic hyperlactatemia causes confusion and affects incidence and prevalence rates.

Nevertheless, it has been hypothesized that a lactate measurement > 5 mmol/L is defined as severe hyperlactatemia and therefore includes lactic acidosis [17,35]. Fabian et al. in South Africa [35] reported a prevalence of severe hyperlactatemia of 1.3% corroborating our 1.1% even though a huge number of their patients were on stavudine-based regimens.

Unfortunately, the cross-sectional design of the present study could not permit us to determine the incidence and time to onset of hyperlactatemia. Menezes et al. [29] reported a median time to the development of lactic acidosis of 10.8 months while Wester et al. [20] reported a median time of 7.5 months. Moreover we were unable to investigate for other risk factors incriminated or not in the development of hyperlactatemia such as higher baseline BMI (> 30 kg/m^2^), lower initial CD4+ T-cells count, higher baseline serum creatinine levels at ART-initiation, this because these informations were lacking in the majority of our patients’ files. It is unclear whether and what extent symptomatic hyperlactatemia might progress to lactic acidosis or whether they are two completely separate disorders [7,37]. In fact, routine monitoring of serum lactate levels does not appear to be warranted [7]. Mild, asymptomatic elevations in lactate levels have not been predictive of progression to lactic acidosis [4,7], and a positive predictive value of only 39% for the confirmation of lactic acidosis following a single elevated value has been reported [37]. Taking into account that lactic acidosis can present quite precipitously and progress in a fulminant manner and those patients can be relatively asymptomatic even with high lactate concentrations, lactate levels should be measured as soon as clinical manifestations suggestive of possible symptomatic hyperlactatemia occur in patients currently taking ART. Marceau et al. [32] have proposed that lactate measurements might be performed every 6 months under standardized conditions in asymptomatic patients to detect a trend of increasing lactate concentrations.

## Conclusion

Our results showed that chronic compensated and moderated hyperlactatemia is common in ART-exposed HIV infected Cameroonians. Even though stavudine is no more part of ART-regimens, being on a first line regimen constitutes a strong and important risk factor for developing hyperlactatemia. Early diagnosis of symptomatic hyperlactatemia by measurement of plasma lactate under standardized conditions may allow safe outpatient care and optimize the management of these HIV-infected patients on ART.

## Competing interest

There is no conflict of interest regarding any of the authors.

## Authors’ contributions

Study Concept and design: JNY, VJAM, CTM, CAP, CT. Data collection and performing experiments: CTM, VJAM. Statistical Analysis: JRNN. Drafting: JRNN, CTM, CAP. Manuscript Revision: TCM, JRNN, VJAM, CAP, CT, JNY. Study Supervision: JNY. All authors read and approved the final manuscript.
